# Supplementary material for: Cyclophilin D knockout significantly prevents HCC development in a streptozotocin-induced mouse model of diabetes-linked NASH
Source: PLoS One. 2024 Apr 4;19(4):e0301711. doi: 10.1371/journal.pone.0301711 (PMC10994289; doi:10.1371/journal.pone.0301711)
Supplement: Attachment — Submitted filename: Response to Reviewers.docx [file pone.0301711.s001.docx]

Dear PLOS One Editors and Reviewers:

We thank the editors and reviewers for taking the time to again read and comment on our manuscript. Below are each of the journal requirements and reviewer comments with our responses following in blue:

Journal Requirements:

Please review your reference list to ensure that it is complete and correct. If you have cited papers that have been retracted, please include the rationale for doing so in the manuscript text, or remove these references and replace them with relevant current references. Any changes to the reference list should be mentioned in the rebuttal letter that accompanies your revised manuscript. If you need to cite a retracted article, indicate the article’s retracted status in the References list and also include a citation and full reference for the retraction notice.

Thank you, we have checked that the reference list is correct and does not include any retracted papers.

[Note: HTML markup is below. Please do not edit.]

Reviewers' comments:

Reviewer's Responses to Questions

**Comments to the Author**

1. If the authors have adequately addressed your comments raised in a previous round of review and you feel that this manuscript is now acceptable for publication, you may indicate that here to bypass the “Comments to the Author” section, enter your conflict of interest statement in the “Confidential to Editor” section, and submit your "Accept" recommendation.

Reviewer #1: All comments have been addressed

Reviewer #2: (No Response)

Thank you. See below for our responses to “6. Review Comments to the Author”.

2. Is the manuscript technically sound, and do the data support the conclusions?

The manuscript must describe a technically sound piece of scientific research with data that supports the conclusions. Experiments must have been conducted rigorously, with appropriate controls, replication, and sample sizes. The conclusions must be drawn appropriately based on the data presented.

Reviewer #1: Yes

Reviewer #2: Yes

Thank you.

3. Has the statistical analysis been performed appropriately and rigorously?

Reviewer #1: Yes

Reviewer #2: I Don't Know

Thank you for your comments. See below for our responses to “6. Review Comments to the Author”.

4. Have the authors made all data underlying the findings in their manuscript fully available?

The [PLOS Data policy](http://www.plosone.org/static/policies.action#sharing) requires authors to make all data underlying the findings described in their manuscript fully available without restriction, with rare exception (please refer to the Data Availability Statement in the manuscript PDF file). The data should be provided as part of the manuscript or its supporting information, or deposited to a public repository. For example, in addition to summary statistics, the data points behind means, medians and variance measures should be available. If there are restrictions on publicly sharing data—e.g. participant privacy or use of data from a third party—those must be specified.

Reviewer #1: Yes

Reviewer #2: Yes

Thank you.

5. Is the manuscript presented in an intelligible fashion and written in standard English?

PLOS ONE does not copyedit accepted manuscripts, so the language in submitted articles must be clear, correct, and unambiguous. Any typographical or grammatical errors should be corrected at revision, so please note any specific errors here.

Reviewer #1: Yes

Reviewer #2: Yes

Thank you.

6. Review Comments to the Author

Please use the space provided to explain your answers to the questions above. You may also include additional comments for the author, including concerns about dual publication, research ethics, or publication ethics. (Please upload your review as an attachment if it exceeds 20,000 characters)

Reviewer #1: This reviewer is not a specialist in liver physiology or tumor formation. The presented results are sound and data are displayed in a proper manner.

CypD is a mitochondrial component - it would be good to present any mechanistical explanations of the observed effects on CypD in tumorigenesis It would be good to supplement these nice observational studies with some experiments aiming on elucidation of the molecular mechanism(s). Or at least separate the mitochondrial effects on MPTP CypD from other members of Pro-isomerases.

Thank you for your comments. As you note and as we go over in the discussion section of the manuscript, CypD is a part of the complex forming the mPTP and thus is known under varying conditions to regulate mitochondrial permeability. However, we mention CypD is part of the mPTP only to discuss obvious avenues for future investigation. We do not claim that CypD has an effect on HCC or NAFLD/NASH progression through its role in the mPTP or its localization to mitochondria at all. Figure 5 shows that CypD KO mice have numerous perturbed genes, mostly depressed, related to HCC but mostly not directly related to the mPTP. It is thus likely that CypD KO has wide ranging effects beyond just mitochondrial permeability, only some of which have been previously reported. While future experiments will certainly be warranted to explain the precise mechanism behind CypD and cyclophilin involvement in HCC formation and progression, we believe this study is important enough to report in this journal as is, in order to guide the future studies we suggest. We also agree that isolating the effects of CypD from other cyclophilin family members is important. This study was initially intended to include comparisons with other Cyp KO mice under the same disease model. As noted in the manuscript however, these arms suffered high mortality such that they could not be included in the final comparison.

Reviewer #2: This referee's comments have been largely addressed with notable exception.
In the abstract authors state: "Both CRV431 and NV556 inhibit
several cyclophilin isoforms, among which cyclophilin D (CypD), an essential part of
the mitochondrial permeability transition pore (mPTP) complex, has not been
previously investigated in this context."
I believe this statement needs to be clarified. As authors would probably agree this study doesn't really investigate mPTP but rather reports changes associated with CypD KO with no direct experiments focused on mPTP. With this respect mention of mPTP in the abstract as "has not been previously investigated in this context" is misleading, since similar to the previous studies, this study doesn't address it either. As such, mPTP "possibility" belongs to the discussion rather than to the abstract section.

Thank you for your valuable input. We agree with your concern and we have edited the abstract to remove mention of the mPTP, since as you note, it is not addressed in this study.

7. PLOS authors have the option to publish the peer review history of their article ([what does this mean?](https://journals.plos.org/plosone/s/editorial-and-peer-review-process#loc-peer-review-history)). If published, this will include your full peer review and any attached files.


If you choose “no”, your identity will remain anonymous but your review may still be made public.


**Do you want your identity to be public for this peer review?** For information about this choice, including consent withdrawal, please see our [Privacy Policy](https://www.plos.org/privacy-policy).

Reviewer #1: No

Reviewer #2: No

Thank you again to all the PLOS One Editors and the reviewers for your comments and improvements to the overall manuscript. The article has been further enriched by your contributions. Please contact the corresponding author(s) if you have any further questions or comments.

Sincerely,

Philippe Gallay

Professor
Department of Immunology & Microbiology 
The Scripps Research Institute
10550 North Torrey Pines Road
La Jolla, California 92037
Tel. (858) 784 8180
Fax (858) 784 8831
<http://www.scripps.edu/gallay/>
